# Dual-energy CT in sacral fragility fractures: defining a cut-off Hounsfield unit value for the presence of traumatic bone marrow edema in patients with osteoporosis

**DOI:** 10.1186/s12891-022-05690-2

**Published:** 2022-07-29

**Authors:** Jan-Peter Grunz, Lukas Sailer, Patricia Lang, Simone Schüle, Andreas Steven Kunz, Meinrad Beer, Carsten Hackenbroch

**Affiliations:** 1 Department of Radiology, German Armed Forces Hospital Ulm, Oberer Eselsberg 40, 89081 Ulm, Germany; 2 Department of Diagnostic and Interventional Radiology, University Hospital Würzburg, Oberdürrbacher Straße 6, 97080 Würzburg, Germany; 3 Department of Orthopedics and Trauma Surgery, German Armed Forces Hospital Ulm, Oberer Eselsberg 40, 89081 Ulm, Germany; 4 Department of Radiology, University Hospital Ulm, Albert-Einstein-Allee 23, 89081 Ulm, Germany

**Keywords:** Dual-energy computed tomography, Fragility fracture, Bone bruise, Bone marrow edema, Virtual non-calcium imaging

## Abstract

**Background:**

Demographic change entails an increasing incidence of fragility fractures. Dual-energy CT (DECT) with virtual non-calcium (VNCa) reconstructions has been introduced as a promising diagnostic method for evaluating bone microarchitecture and marrow simultaneously. This study aims to define the most accurate cut-off value in Hounsfield units (HU) for discriminating the presence and absence of bone marrow edema (BME) in sacral fragility fractures.

**Methods:**

Forty-six patients (40 women, 6 men; 79.7 ± 9.2 years) with suspected fragility fractures of the sacrum underwent both DECT (90 kVp / 150 kVp with tin prefiltration) and MRI. Nine regions-of-interest were placed in each sacrum on DECT-VNCa images. The resulting 414 HU measurements were stratified into “edema” (*n* = 80) and “no edema” groups (*n* = 334) based on reference BME detection in T2-weighted MRI sequences. Area under the receiver operating characteristic curve was calculated to determine the desired cut-off value and an associated conspicuity range for edema detection.

**Results:**

The mean density within the “edema” group of measurements (+ 3.1 ± 8.3 HU) was substantially higher compared to the “no edema” group (-51.7 ± 21.8 HU; *p* < 0.010). Analysis in DECT-VNCa images suggested a cut-off value of -12.9 HU that enabled sensitivity and specificity of 100% for BME detection compared to MRI. A range of HU values between -14.0 and + 20.0 is considered indicative of BME in the sacrum.

**Conclusions:**

Quantitative analysis of DECT-VNCa with a cut-off of -12.9 HU allows for excellent diagnostic accuracy in the assessment of sacral fragility fractures with associated BME. A diagnostic “one-stop-shop” approach without additional MRI is feasible.

## Background

Fragility fractures are induced by an acute trauma that would be insufficient to fracture a bone with normal microarchitecture [1]. Accordingly, this fracture entity is associated with alterations to bone quality with osteoporosis being considered the main risk factor [2]. For instance, Oberkircher et al. found that more than 60% of pelvic ring fractures in the elderly were associated with osteoporosis [3]. Demographic changes warrant a further increase in the incidence of fragility fractures of the sacrum (FFS) [4], which is also reflected by the introduction of a dedicated classification system by Rommens et al. in 2012 [5]. Conventional CT and MRI are the main established methods used for diagnosing FFS, according to the ACR appropriateness criteria [6]. While CT is well suited to depict the osseous anatomy and in particular cortical fracture lines, MRI bears the advantage of visualizing the bone marrow, apart from not relying on radiation exposure. At present, MRI has the highest sensitivity for detecting FFS, which, by contrast, may be occult in conventional CT scans [7–10]. In addition to offering a wide range of applications from urology over rheumatology and musculoskeletal radiology [11], dual-energy CT (DECT) has been reported to allow for visualizing bone marrow edema (BME), e.g., regarding the spine [12], hip [13], knee [14], and ankle [15]. Hereby, the virtual non-calcium (VNCa) technique enables subtraction of calcium from cancellous and cortical bone, rendering the bone marrow isolated for assessment – a former domain of MRI [16]. Previous studies have shown DECT to be superior to conventional CT and as effective as MRI in detecting fragility fractures of the pelvic ring [17].

Dual-energy X-ray absorptiometry constitutes the current method of choice for measurement of bone mineral density (BMD) [18]. Although quantitative CT was introduced prior to dual-energy X-ray absorptiometry and provides excellent results, it cannot rival its extremely low radiation dose [19]. However, so-called “asynchronous quantitative CT” allows for BMD assessment, too, using CT scans obtained for other indications. Jang et al. measured the BMD in the cancellous bone of the first lumbar vertebra in routine CT, suggesting this to offer a simple reference for patients at risk for osteoporosis [20]. Another promising approach to measure BMD has been described for DECT [21, 22].

The primary objective of this study was to define the cut-off value of Hounsfield units (HU) with the highest accuracy for discriminating between the presence and absence of FFS in DECT scans. A second objective was to assess whether osteoporosis can be detected by means of DECT based on region-of-interest (ROI) measurements.

## Methods

The study and all scan protocols were approved by the institutional review board of the University of Ulm (IRB number: 343/16) and registered in the German Clinical Trials Register (DRKS00010552). The need for additional written informed consent was waived by the local ethics committee.

### Patient population and inclusion/exclusion criteria

This retrospective investigation included patients who presented to our department with suspected FFS over a period of thirty months between December 2015 to June 2018. Inclusion criteria demanded a patient age of 55 years or more, as well as availability of DECT and MRI scans. Patients presenting with high-energy trauma or any systemic disease affecting the bone marrow were excluded. A total of 46 individuals constituted the final study cohort (40 women, 6 men, mean age: 79.7 ± 9.2 years). Mean intervals between DECT and MRI were calculated at 2 ± 2 days (range 0 – 4 days). The same population has been investigated previously in a study comparing the diagnostic abilities of DECT and conventional CT [17].

### Scan protocols and technical details

All DECT scans were performed on a third-generation dual-source CT (Somatom Force, Siemens Healthineers, Erlangen, Germany). Standard protocols for spinal DECT scans were modified for pelvic imaging, setting tube voltages at 90 kV (200 reference mAs) and 150 kV with tin prefiltration (125 reference mAs). Scan parameters further included collimation of 128 × 0.6 mm, helical pitch factor of 0.6, and gantry rotation time of 0.5 s. For dual-energy post-processing, reconstructions in coronal orientation were prepared with a medium-soft convolution kernel (Qr40) and level 3 of an iterative reconstruction algorithm in dedicated post-processing software (syngo.via VB10A, Siemens Healthineers). VNCa images of the pelvis were reconstructed in coronal orientation. BME was depicted in two different image sets, i.e., images color-coded in green and blue and images resembling the monochromatic impression of MRI scans. During the study period, three different MRI scanners were used for diagnostic purposes at our institution, i.e., a 1.0-Tesla unit (Magnetom Harmony, Siemens Healthineers), a 1.5-Tesla unit (Philips Medical Systems, Best, Netherlands), and a 3.0-Tesla unit (Achieva, Philips Medical Systems). Presence and extent of BME was assessed on the basis of standard fluid-sensitive fat-saturated sequences (T2 TIRM / T2 STIR / T2 SPAIR; TR 3800–9250, TE 45–80). No intravenous contrast agents were applied for any of the scans performed in this study.

### “Edema” and “no edema” groups

**Fig. 1:**
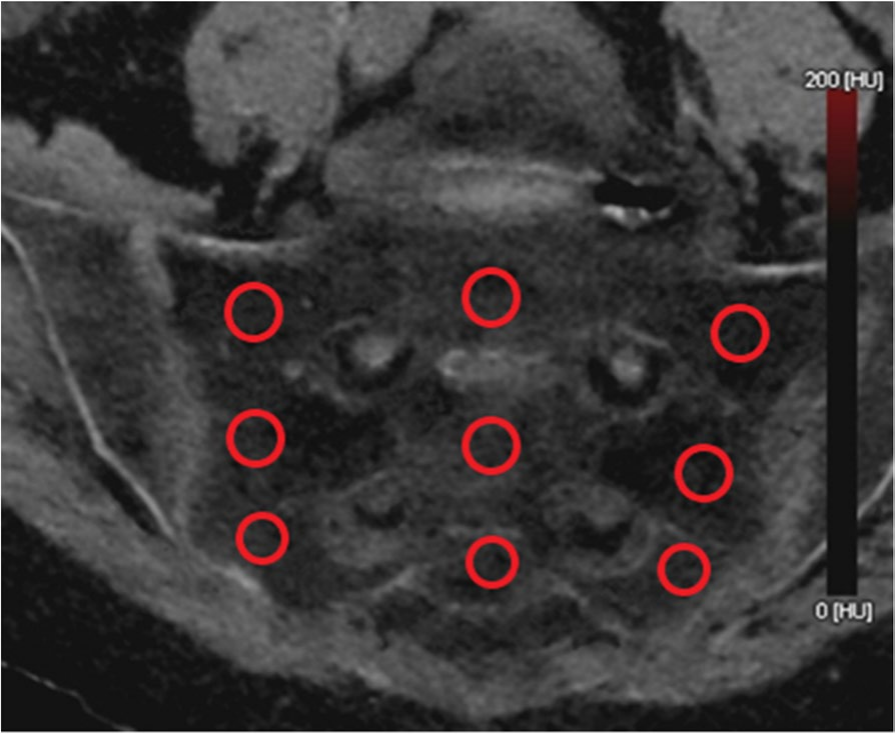
Virtual non-calcium (VNCa) image of the sacrum (coronal view). The red circles represent the nine locations where Hounsfield units (HU) were measured within regions of interest

MRI served as reference standard for diagnosis of BME, with two radiologists analyzing each study in consensus reading. Hereby, the presence of edema was assessed in dichotomous fashion (present/absent). HU values were measured on the monochromatic VNCa images by means of ROI analysis in nine specific locations within each sacrum (Fig. 1). To account for the geometrical differences between patients, each set of ROIs was placed individually by a radiologist with more than 5 years of clinical CT experience. Care was taken to ensure that ROIs were placed within bone marrow and not over cortical bone, areas of sclerosis or adjacent soft tissue. The “edema” group included all DECT measurements obtained at locations for which MRI scans indicated the presence of BME. Measurements at locations where MRI did not depict edema were assigned to the “no edema” group.

### “Fracture and osteoporosis”, “fracture and suspected osteoporosis”, and “no fracture” groups

The second study arm aimed to define a range of density values that allow for osteoporosis to be suspected based solely on DECT. For this purpose, additional ROIs were placed in the cancellous bone of the fourth lumbar vertebra (L4) or alternatively the fifth lumbar vertebra (L5) if bone density at L4 could not be calculated, e.g., due to previous fractures. The prevalence of osteoporosis was assessed within the study population and three groups were established for analysis: The “fracture and osteoporosis” group included all measurements from patients with both FFS and established diagnosis of osteoporosis (via dual-energy X-ray absorptiometry or quantitative CT), whereas individuals without an established diagnosis of osteoporosis but with a typical FFS were assigned to the “fracture and suspected osteoporosis” group. Finally, the “no fracture” group comprised all HU measurements from patients without evidence of an osteoporotic fracture.

### Statistical analysis

All analyses were performed with dedicated spreadsheet (Excel, Microsoft, Redmond, Washington, USA) and statistical software (QuickCalcs, GraphPad Software, San Diego, California, USA). Calculation of the area under the receiver operating characteristic curve (AUC-ROC) was used to evaluate the HU values derived from dual-energy VNCa images and to determine the cut-off value with the most accurate discrimination for presence or absence of BME. For this analysis, MRI served as the standard of reference. Employing the resulting cut-off value, sensitivity and specificity were computed. Comparison of parametric, normally distributed variables was performed using student’s t-tests. Statistical significance was established at an alpha level of 0.05.

## Results

### Comparison of “edema” and “no edema” groups

**Fig. 2:**
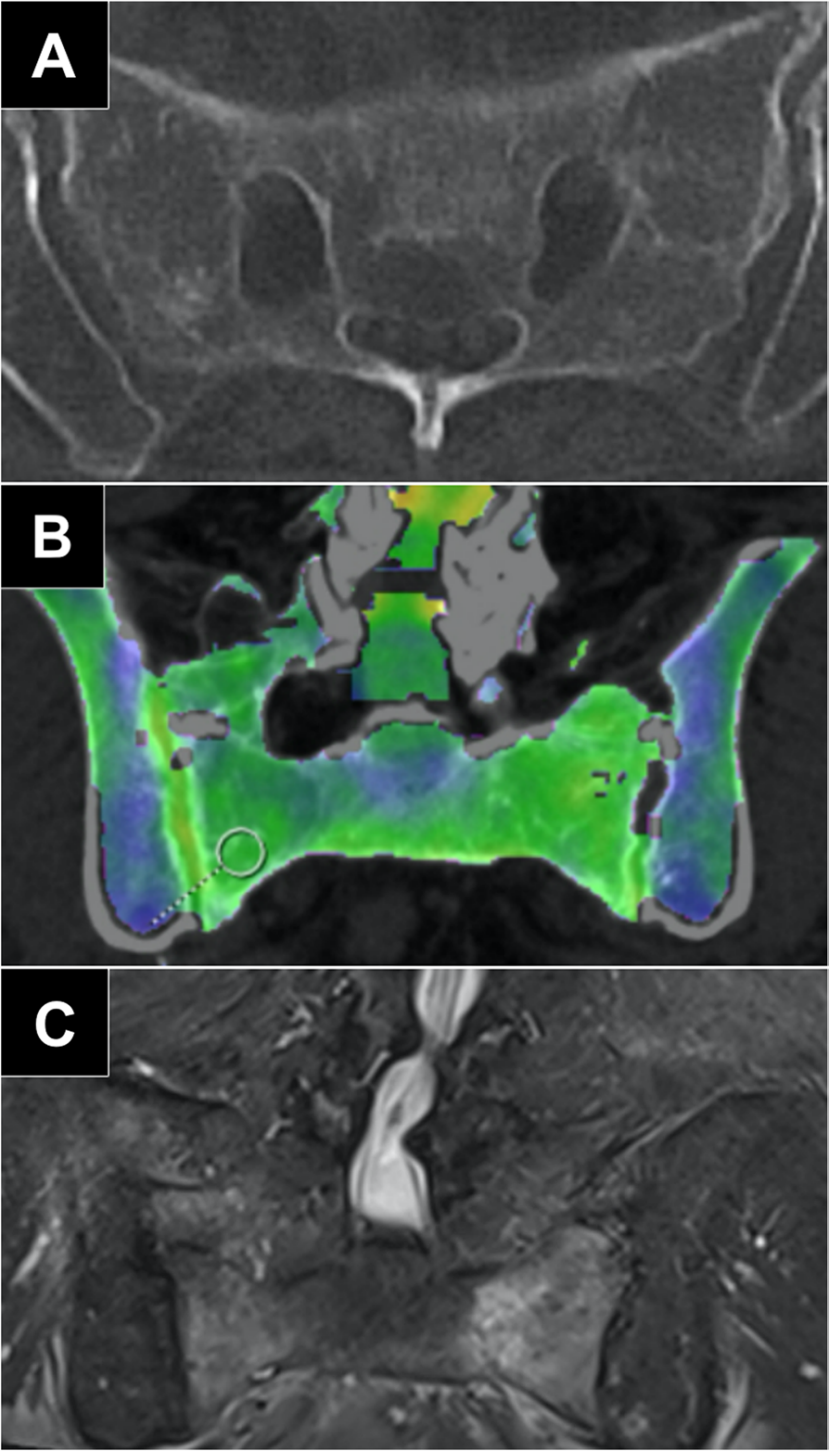
**a** Axial CT image that fails to demonstrate a discrete fracture line in the sacrum. **b** Color-coded coronal DECT reconstruction image with region-of-interest measurement in the right inferior region of the sacrum. Blue represents lower HU values of approximately -50 HU, whereas yellow/light green indicates higher HU values around + 10 HU. **c** Coronal T2-weighted STIR sequence demonstrates edema in the same locations

**Fig. 3:**
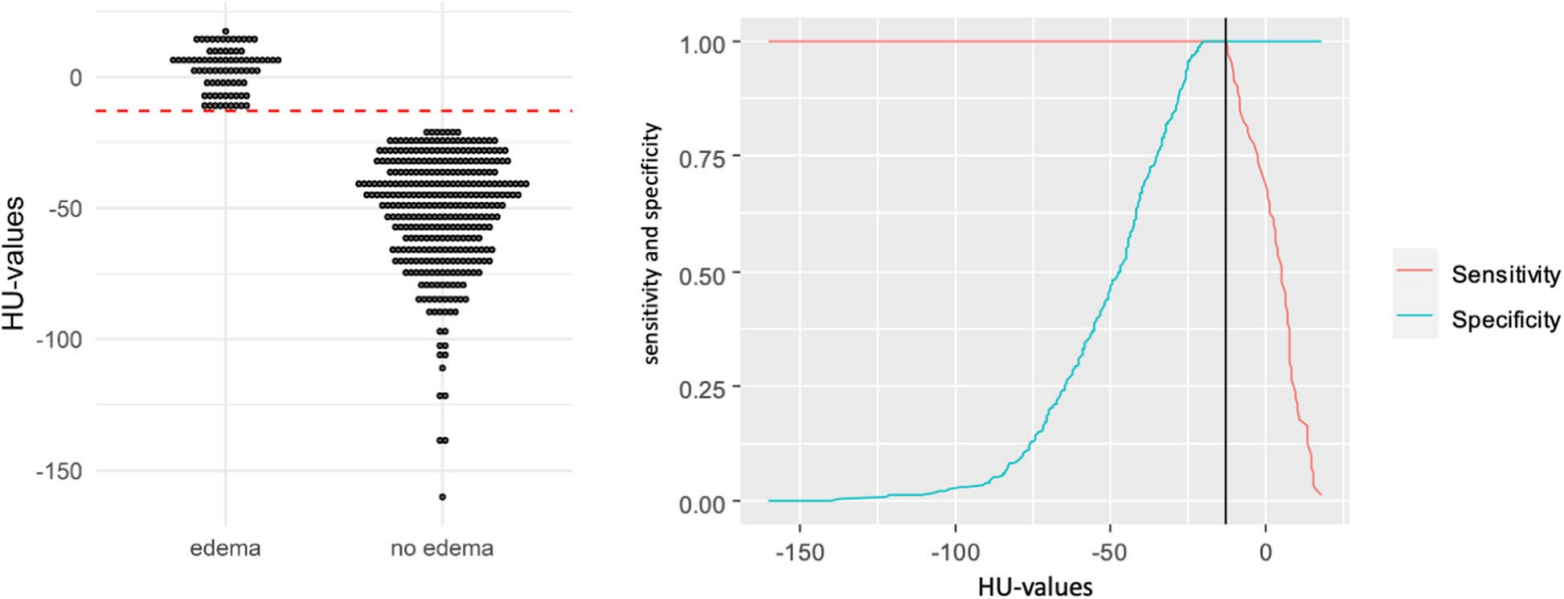
Dotplots showing the distribution of Hounsfield unit (HU) measurements using dual-energy CT. The red dotted line indicates the calculated cut-off value of -12.9 Hounsfield units (HU), which allowed for the most accurate discrimination between the presence or absence of bone marrow edema (left). Employing the cut-off value to calculate classification functions of diagnostic accuracy results in the highest possible sensitivity and specificity (right)

The “edema” group was comprised of 80 measurements, associated mean density was + 3.1 ± 8.3 HU (range: -12.9 to + 17.6 HU). In contrast, the “no edema” group consisted of a total of 334 measurements. Within this cohort, the mean ROI-based value was found to be -51.7 HU ± 21.8 HU (range: -160.0 to -20.1 HU). Figure 2 provides an exemplary patient’s case of BME missed in standard CT but detected in VNCa. The measured density within the “edema” group was substantially higher compared to the “no edema” group (*p* < 0.010). Based on the measurement results, we calculated a range of HU values that suggest the presence of BME on DECT scans according to the formula “*(mean* ± standard deviation) * 2”. Derived from this calculation, HU values ranging from -14.0 to + 20.0 are reported to be indicative of BME. AUC-ROC analysis of mean DECT numbers in VNCa images suggested a cut-off value of -12.9 HU that allowed for both sensitivity and specificity of 100% for BME (Fig. 3).

### Comparison of “fracture and osteoporosis”, “fracture and suspected osteoporosis”, and “no fracture” groups

**Fig. 4:**
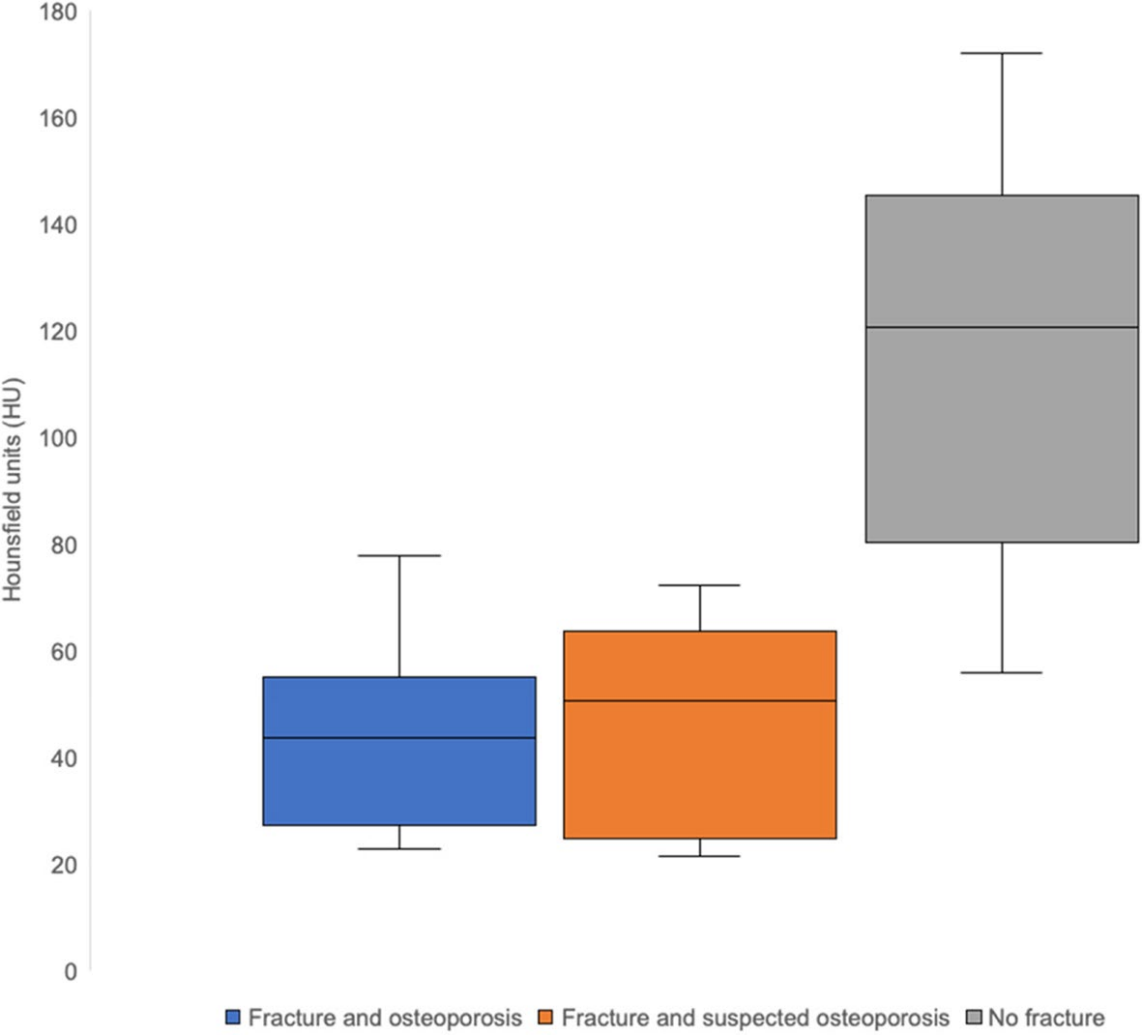
Box-and-whisker plot showing the distribution of Hounsfield unit (HU) measurements at the fourth or fifth lumbar vertebra in patients with osteoporosis (“fracture and osteoporosis”), patients without osteoporosis but with osteoporotic fractures (“fracture and suspected osteoporosis”) and patients without osteoporosis and without osteoporotic fractures (“no fracture”)

Individuals with an established diagnosis of osteoporosis accounted for 22 of 46 patients (47.8%) in our study. Within the “fracture and osteoporosis” group, the mean value of ROI measurements within cancellous bone of lumbar vertebral bodies was 43.9 ± 16.9 HU (range: 22.7–77.7 HU). The “fracture and suspected osteoporosis” group included measurements from 13 patients without established osteoporosis who were diagnosed with a typical osteoporotic fracture during hospitalization. The mean density value in this group was 47.0 ± 18.4 HU (range: 21.5–72.3 HU). Eleven individuals were included in the “no fracture” group with a calculated a mean density value of 115.2 ± 36.9 HU (range: 55.9–172 HU). The mean density within the “fracture and osteoporosis” group was considerably lower compared to the “no fracture” group (*p* < 0.001). Similarly, the mean density within the “fracture and suspected osteoporosis” group was substantially lower compared to the “no fracture” group (*p* < 0.001). By contrast, the difference between the “fracture and osteoporosis” and “fracture and suspected osteoporosis” cohorts did not differ significantly (*p* = 0.340). Figure 4 provides detailed ROI-based density measurement results.

## Discussion

In this retrospective single-center study on detection of sacral fragility fractures in dual-energy CT, we were able to show that density values at locations with associated edema differed significantly from those without edema. We concluded that a range of -14.0 to + 20.0 HU in virtual non-calcium images is indicative of the presence of bone marrow edema and that CT numbers outside of this range render an acute fracture highly unlikely. Notably, a cut-off value of -12.9 HU provides the most accurate discrimination between edema presence or absence.

Our measurements of bone density revealed a significant difference between patients with typical osteoporotic fractures and established / suspected diagnosis of osteoporosis as compared to patients without suchlike fractures (43.0 HU / 47.0 HU vs. 115.2 HU). This finding is supported by the results of Schreiber et al. who described a significant correlation between T-scores of dual-energy X-ray absorptiometry and BMD assessment by CT [23]. Correspondingly, Pickhardt et al. compared density values and T-scores in 1867 patients and found that an attenuation of 160 HU or less at the first lumbar vertebra was 90% sensitive and a threshold of 110 HU was more than 90% specific for distinguishing osteoporosis [24]. DECT bears the advantage of combining the benefits of conventional CT in evaluating osseous structures with those of MRI in assessing bone marrow in a single imaging modality [17]. This is of particular importance as FFS can be difficult to diagnose on conventional CT scans and may potentially remain occult since they are often characterized by minimal cancellous fracture lines and BME and not necessarily by cortical disruption [8, 9]. Thus, DECT may be able to serve as a “one-stop-shop” approach to fragility fracture analysis, preserving resources and avoiding additional and lengthy MRI examinations. Also, in case of contraindications to MRI, DECT promises to be a comprehensive alternative.

**Fig. 5:**
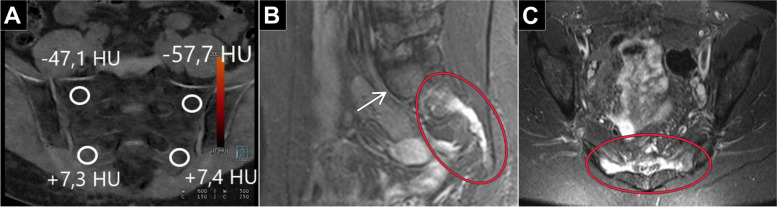
**a** Coronal VNCa reformatting of dual-energy CT suggested the absence of edema at the upper measurement sites (-47.1 HU and -57.7 HU), whereas the presence of edema was indicated for the lower measurement sites (+ 7.3 HU and + 7.4 HU). **b** Corresponding sagittal T2( STIR image (at 1.5 Tesla) of the same patient with marked edema in the lower sacrum (red circle). In contrast, no edema was ascertained in the upper sacrum (white arrow). **c** Axial T2 STIR image (at 1.5 Tesla) confirmed the presence of edema in both sides of the lower sacrum (red circle)

While color-coded presentation of VNCa datasets may sometimes be sufficient for visualization of BME, a quantitative approach has been shown to facilitate a more accurate assessment on fracture presence, while allowing to locate the site and extent of an acute FFS [25]. Figure 5 depicts a patient with a fragility fracture limited to the lower sacrum, while the upper portion of the bone is merely osteoporotic. In that case, adherence to the calculated cut-off value of -12.9 HU and the associated conspicuity range of -14.0 to + 20.0 HU allowed for correct distinction of regions with BME. Notably, the CT numbers reported in this study are in line with the findings of Bierry et al., who reported cut-off values of 35 HU for the thoracic and 6.5 HU for the lumbar spine [26]. Petritsch et al. (-47 HU) and Wang et al. (-80 HU) proposed lower cut-off values for compression fractures of the spine without differentiating between lumbar and thoracic vertebrae. Furthermore, these studies did not consider age-related factors, such as bone marrow composition and osteoporosis in the investigation [12, 27]. In a recent study with particular focus on sacral insufficiency fractures, Booz et al. also suggested a lower threshold (-43 HU). Notably, however, the investigated cohort comprised of considerably younger patients and less women compared to the population included in the present work (61 ± 13 years, 54% females versus 80 ± 9 years, 87% females), which presumably explains the diverging results [28].

### Limitations

Some limitations of the study need to be acknowledged. The reported results are based solely on findings regarding the sacrum as other osseous structures of the pelvis offer only a limited bone marrow volume in most cases. While the proposed density range indicating BME appears promising, the size of the study group and the focus on a solitary dual-source CT model with optimized spectral separation may limit transferability of results. Hence, further investigations are warranted, particularly with the emergence of novel photon-counting detector systems. Lastly, careful patient selection is mandatory when performing DECT for FFS, since detection of BME is superior in elderly patients with a higher percentage of fatty marrow as opposed to younger patients with more dense trabecular bone [27]. In addition to this population, we consider MRI superior in cancer patients with bone or soft tissue metastases, and in patients with neurological symptoms that suggest neuroforaminal affection.

## Conclusion

Comprehensive diagnosis of fragility fractures of the sacrum can be achieved by dual-energy CT in terms of a “one-stop-shop approach”. We postulate that a range of density values from -14 to + 20 HU within bone marrow in virtual non-calcium images indicates associated edema, with the most accurate cut-off value defined at -12.9 HU. Additional assessment of bone density at the fourth and fifth lumbar vertebra can indicate the presence of osteoporosis, which constitutes the main risk factor for fragility fractures of the sacrum. In this regard, CT numbers below 80 HU should raise increased awareness for fragility fractures.

## Data Availability

The datasets generated and/or analyzed during this study are not publicly available as CT data and DICOM headers contain patient information. Data can be obtained on reasonable request from the corresponding author.

## Abbreviations

BMD: Bone mineral density
BME: Bone marrow edema
DECT: Dual-energy computed tomography
FFS: Fragility fractures of the sacrum
ROI: Region of interest
VNCa: Virtual non-calcium
